# Effects of Eye Movement Training and Changes of Gaze During Walking in a Patient With Oculomotor Disorder After Brainstem Hemorrhage

**DOI:** 10.7759/cureus.77576

**Published:** 2025-01-17

**Authors:** Miku Kunoh, Daisuke Kimura, Kenta Kunoh, Kazumasa Yamada

**Affiliations:** 1 Department of Rehabilitation, Yamada Hospital, Gifu, JPN; 2 Department of Occupational Therapy, Faculty of Medical Sciences, Nagoya Women’s University, Nagoya, JPN; 3 Faculty of Rehabilitation Sciences, Aichi Medical College, Nagakute, JPN

**Keywords:** eye movement, gaze, oculomotor disorder, stroke, walking

## Abstract

We report a case of a patient who developed pontine hemorrhage and presented with eye movement disorders but was able to regain conjugate eye movement through eye movement training, resulting in improved walking ability.

The patient was a 39-year-old man who presented with cerebral hemorrhage. He was admitted to the hospital due to a pontine hemorrhage extending from the midbrain to the medulla oblongata and perforation of the fourth ventricle. Symptoms included right hemiplegia, right upper and lower limb paresthesias, ataxia of the trunk, left abducens nerve palsy, left facial nerve palsy, right oculomotor nerve palsy, right trochlear nerve palsy, right Horner's syndrome, and longitudinal nystagmus. From the 121st day, eye movement training was performed for five days per week for 10 weeks to treat oculomotor dysfunction.

For eye movement evaluation, left and right eye movements during pursuit eye movement, which involved following the contours of a figure, and during walking were measured with an eye movement measuring device (eye camera) (TalkEye Light; Takei Kiki Co. Ltd., Japan). In addition, motor function assessment included ataxia, lower limb muscle strength, physical balance function, and walking ability. Measurements were taken before the start of the eye movement training, two weeks after walking ability improved, and then at 10 weeks.

After 10 weeks of eye movement training, the range of motion of the eyeballs during pursuit eye movement was expanded, and both eyes moved in the same direction and by the same amount. The eyes moved similarly to those of a healthy subject during walking two weeks and 10 weeks after the start of eye movement training, when walking ability improved, the left and right gazes overlapped, and both eyes were focused on the center of the forward visual field. Motor function improved in all categories.

The eye movement training improved eye movements, and strabismus and diplopia were no longer observed. We suggest that eye movement training, in addition to conventional motor training, may be a means to improve walking ability in stroke patients in order to obtain a stable gait.

## Introduction

Factors related to gait after stroke include motor (muscle strength and motor paralysis), sensory, and cognitive functions [1-3], and visual function is important for a stable gait [4,5]. Regarding visual function, visual impairments after brain injury have been reported [6-8], following 60% of stroke population [9]. Visual impairment after stroke leads to an impact on daily life. In stroke survivors with visual impairment, the effects of vision problems have been reported to include loss of confidence, being a burden to others, increased collisions/accidents, and fear of falling [10].

In sports, there have been many reports on the effectiveness of training for improving visual function [11-14]. However, there have been few studies on eye movement training in patients with stroke and elderly patients, and there are only a few reports on the effects of eye movement training on visual function in these patient populations.

In this study, we report a case of a patient who developed pontine hemorrhage and presented with eye movement disorders but was able to regain conjugate eye movement through eye movement training, resulting in improved walking ability.

## Case presentation

**Figure 1:**
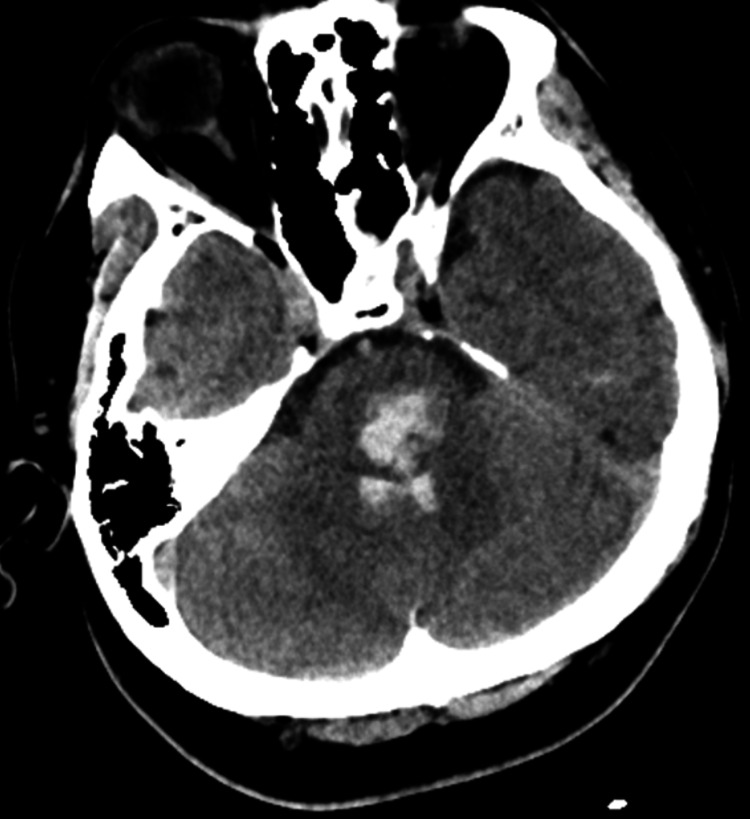
Diagnostic findings of intracerebral hemorrhage visualized through brain imaging

The patient, a 39-year-old man, presented with cerebral hemorrhage. He experienced numbness throughout his body while driving a car and was rushed to a nearby hospital. Symptoms included right hemiparesis, right upper and lower limb paresthesia, trunk ataxia, left abducens nerve palsy, left facial nerve palsy, right oculomotor nerve palsy, right trochlear nerve palsy, right Horner's syndrome, and longitudinal nystagmus. He was diagnosed with pontine hemorrhage extending from the midbrain to the medulla oblongata, with perforation of the fourth ventricle, based on head magnetic resonance imaging. He was hospitalized on the same day (Figure 1).

On day 6, dysphagia and respiratory dysfunction were observed, and a tracheotomy was performed, requiring 77 days of intubation. Rehabilitation began on day 10, and on day 56, the patient was transferred to the hospital’s rehabilitation unit. His motor function values at the time of transfer were 3/56 points on the Functional Balance Scale (FBS) and 30/40 points on the Scale for the Assessment and Rating of Ataxia (SARA). He required moderate assistance during standing, transferring, and parallel-bar walking as he was unstable. The patient's higher brain function values were 16/18 points on the Frontal Assessment Battery (FAB), two minutes 36 seconds on the Trail Making Test (TMT) part A, three minutes 50 seconds on the TMT part B, and 25/30 points on the Mini-Mental State Examination (MMSE). Thus, there was no problematic higher brain dysfunction. The Functional Independence Measure (FIM) scores were 25/91 points for the motor items and 22/35 points for cognitive items, and he required assistance with all aspects of daily living. Therefore, a program was implemented to improve the patient’s walking ability. Consequently, the patient’s muscle strength, ataxia, and sensory deficits improved, and he was able to walk using a forearm-supported walker with axillary assistance. When the patient was able to speak after tracheal cannula removal, he complained of difficulty in seeing, saying that since the onset of the disease, he had difficulty walking because of double vision of people and the objects around them. Therefore, eye movement training was started on day 121 of onset. The patient was verbally informed that his case would be reported in the journal, and written consent was obtained.

Eye movement training was performed with the patient in a wheelchair-sitting position, with some modifications to Watabe’s method [15]. The specific details are listed in Table 1. Eye movement training was performed five days per week for 10 weeks (days 121-191).

**Table 1:** Program of eye movement training

Eye movement training		
Pursuit exercise	The patient was instructed to follow the pen nib, which was moved slowly up and down and left and right, with one eye and then with both eyes.	1 minute
Saccade exercise	The patient was instructed to follow the pen nib, which was moved rapidly up and down and left and right, with one eye and then with both eyes.	1 minute
Exercise using the labyrinthine eye reflex	The patient was instructed to keep looking at the fixed nib while moving his head up, down, left, and right.	1 minute
Congestion	The patient was instructed to watch the nib moving back and forth.	1 minute
Typing	The patient was instructed to use the computer to type faster.	10 minutes

**Figure 2:**
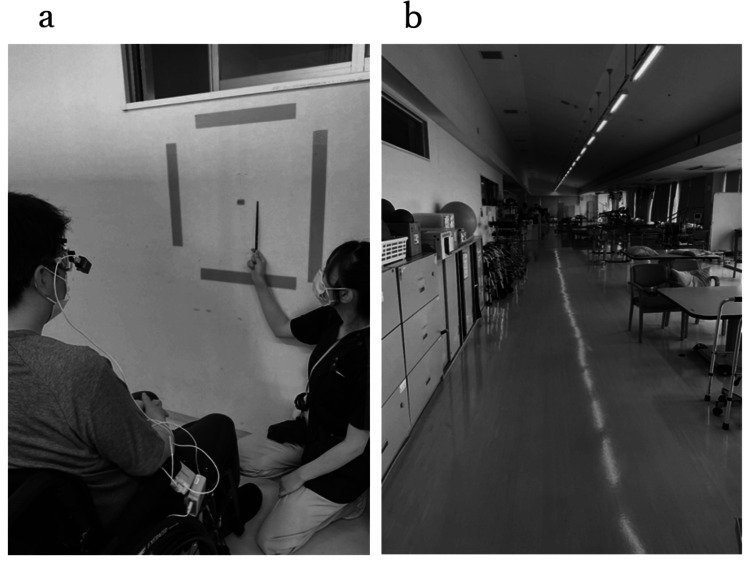
The measurement environments (a) The measurement environment of SPEM; the patient was asked to follow the quadrilateral formed by tape on the wall vertically and horizontally with his eyes. (b) The measurement environment of eye movements during walking; the patient was asked to walk 10 m straight at a comfortable speed in the rehabilitation room. SPEM: smooth pursuit eye movement

For evaluation of eye movements, the left and right eye movement angles were measured at a sampling rate of 30 Hz using an eye movement measuring device (eye camera) (TalkEye Light; Takei Kiki Co. Ltd., Japan). The detection wavelength was 870 nm, and the detection ranges were 50 degrees to the left, 50 degrees to the right, 20 degrees to the top, and 40 degrees to the bottom. The detection resolutions were 0.1 degrees (≤±20 degrees) and 0.5 degrees for the whole area, and the detection errors were <1 degrees (≤±20 degrees), two degrees (≤±40 degrees), and three degrees for the whole area. The evaluation parameters were smooth pursuit eye movement (SPEM) and eye movement during walking. The measurement environments are shown in Figure 2.

For SPEM (Figure 2a), a quadrilateral was created by placing a piece of tape, 56 cm long and 56 cm wide, on the wall of the rehabilitation room. The center of the quadrilateral was placed at eye level with the patient in a seated position in a wheelchair. The wheelchair was placed 1 m away from the wall. The nib was moved around the quadrilateral and crosswise as if tracing a tape. The patient was instructed to follow the nib with his eyes, and an eye camera was used to measure eye movements. The speed of the pen nib was set to the speed at which all tracings were completed within two minutes. Measurements were obtained before and after 10 weeks of eye movement training. Eye movements during walking (Figure 2b) were measured with an eye camera while the patient walked with a forearm-supported walker with bilateral axillary support before the start of eye movement training and with a pick-up walker with a watchful eye after two and 10 weeks of eye movement training. Walking was performed in the rehabilitation room at a comfortable speed for a straight distance of 10 m.

For the evaluation of motor function, SARA was used as an index of ataxia, knee extension muscle strength (determined using a hand-held dynamometer) was used as an index of lower limb muscle strength, FBS and Timed Up and Go Test (TUG) results were used as indices of physical balance function, and 10 m walking time and number of steps were used as indices of walking ability. All procedures were performed before the start of eye movement training, two weeks later, and at the end of 10 weeks; then, the values were compared.

To examine the effectiveness of eye movement training, we compared the eye movement angles before and 10 weeks after the start of eye movement training in SPEM of our patient with the eye movement angles of a healthy male in his 20s (control) with no history of visual or motor function using scatter plots. Additionally, the correlation coefficients and regression lines were calculated for the vertical and horizontal eye movement angles of the left and right eyes to confirm the relationship between the movements of the two eyes. Eye movements of our patient during walking were compared with those of the control using scatter plots of eye movement angles before the start of training, two weeks after the start of training, and 10 weeks after the start of training.

**Figure 3:**
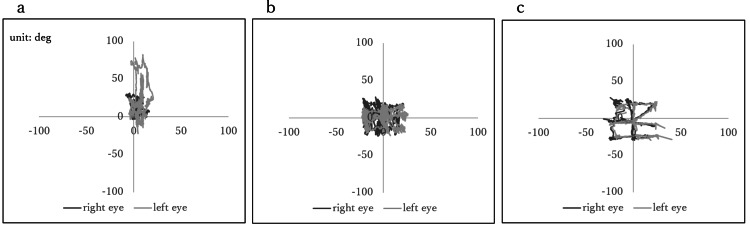
Scatter plots of pursuit eye movement (a) Before the start of eye movement training. (b) After 10 weeks of eye movement training. (c) Control. The horizontal axis indicates the angle in the left-right direction of eye movement, whereas the vertical axis indicates the angle in the upward-downward direction of eye movement. A positive value on the horizontal axis indicates eye movement to the right, whereas a negative value indicates eye movement to the left. A positive value on the vertical axis indicates upward eye movement, whereas a negative value indicates downward eye movement.

Scatter plots of pursuit eye movement are shown in Figure 3. Before the start of eye movement training, the right eye moved -10 to 20 degrees in the horizontal direction and -20 to 80 degrees in the vertical direction; the left eye moved -10 to 20 degrees in the horizontal direction and 0 to 30 degrees in the vertical direction (Figure 3a). After 10 weeks, the right eye moved -20 to 20 degrees laterally and -20 to 20 degrees vertically, and the left eye moved -20 to 20 degrees laterally and -20 to 20 degrees vertically, showing the trajectory of the quadrilateral and crosswise figures on the wall (Figure 3b). Both of the control’s eyes moved in the range of -20 to 20 degrees in the horizontal direction and -20 to 20 degrees in the vertical direction (Figure 3c).

Table 2 shows the relationship between the angles of vertical and horizontal eye movements in the study patient’s left and right eyes. The correlation coefficient and regression line at the beginning of the eye movement training were 0.573 and y=0.46x+5.33 in the horizontal direction and 0.956 and y=2.71x-7.41 in the vertical direction, respectively. The correlation coefficient and regression line at the end of 10 weeks were 0.972 and y=1.18x+3.24 in the left-right direction and 0.943 and y=0.781x+2.47 in the vertical direction, respectively. The correlation coefficient and regression line for the control were 0.991 and y=0.97x+3.24 in the left-right direction and 0.993 and y=0.90x-1.57 in the vertical direction, respectively.

**Table 2:** The relationship between the angles of vertical and horizontal eye movements in the left and right eyes

	Before the start of training	10 weeks later	Control subject
Horizontal direction (Correlation coefficient, regression line)	0.573, y=0.46x+5.33	0.972, y=1.18x+3.24	0.991, y=0.97x+3.24
Vertical direction (Correlation coefficient, regression line)	0.956, y=2.71x-7.41	0.943, y=0.781x+2.47	0.993, y=0.90x-1.57

**Figure 4:**
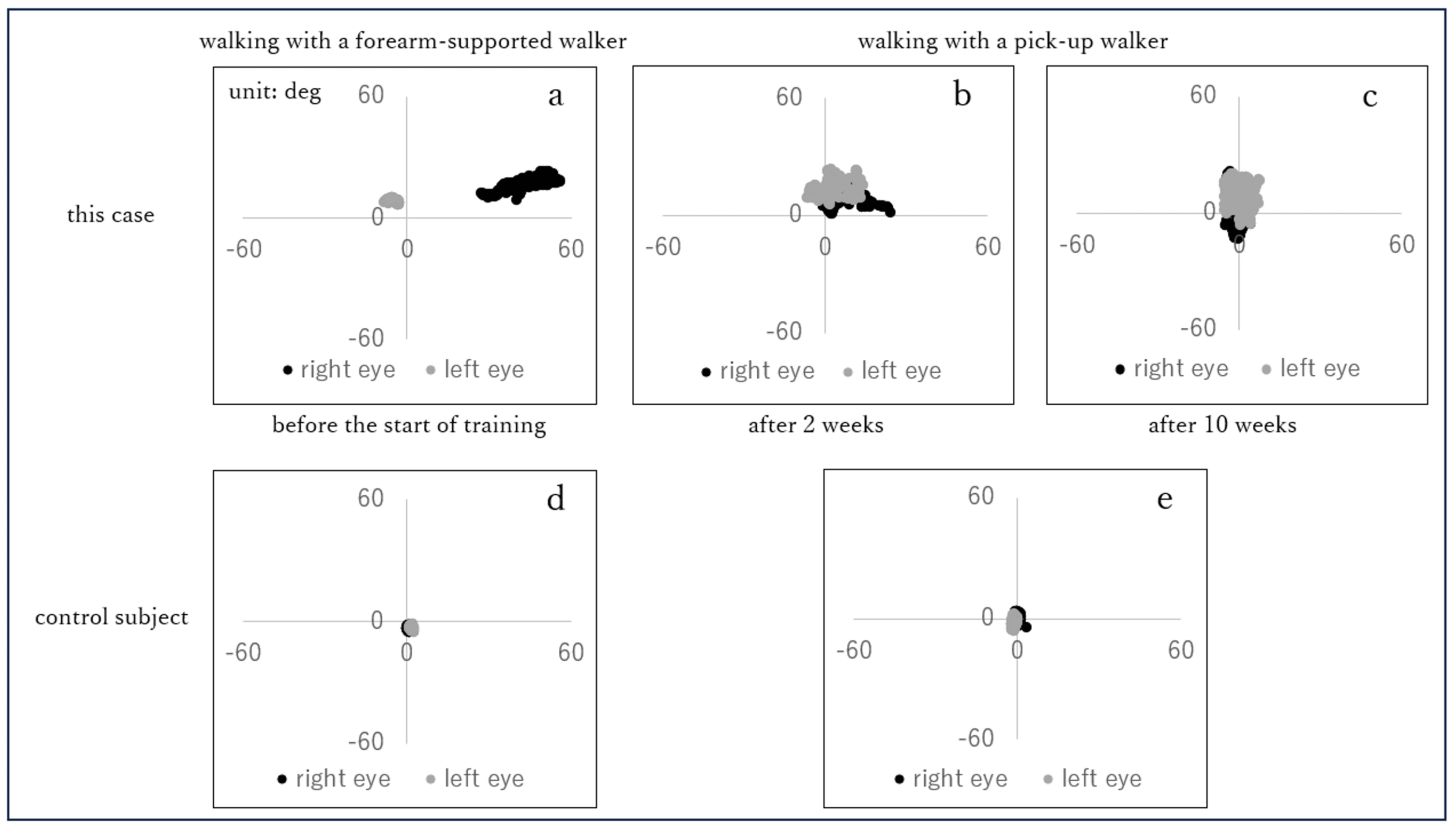
Scatter plots of eye movements during walking (a) Before the start of eye movement training (walking with a forearm-supported walker). (b) After two weeks of eye movement training (walking with a pick-up walker). (c) After 10 weeks of eye movement training (walking with a pick-up walker). (d) Control (walking with a forearm-supported walker). (e) Control (walking with a pick-up walker). The horizontal axis indicates the angle in the left-right direction of eye movement, whereas the vertical axis indicates the angle in the upward-downward direction of eye movement. A positive value on the horizontal axis indicates eye movement to the right, whereas a negative value indicates eye movement to the left. A positive value on the vertical axis indicates upward eye movement, whereas a negative value indicates downward eye movement.

Scatter plots of eye movements during walking are shown in Figure 4. Before the start of eye movement training, the study patient’s right eye moved between -10 and -5 degrees in the horizontal direction and between five and 10 degrees in the vertical direction, whereas the left eye moved between 25 and 60 degrees in the horizontal direction and between 10 and 25 degrees in the vertical direction (Figure 4a). The gaze of the control moved approximately 0 degrees horizontally and -5 to 0 degrees vertically for the right eye, and 0 to five degrees horizontally and -5 to 0 degrees vertically for the left eye (Figure 4d). After two weeks of eye movement training, the study patient’s right eye was moving in the range of -1 to 24 degrees laterally and one to 19 degrees vertically; the left eye was moving in the range of -7 to 14 degrees laterally and six to 24 degrees vertically. The images of both eyes were overlapping (Figure 4b). After 10 weeks of eye movement training, the study patient’s right eye was moving in the range of -5 to five degrees laterally and -15 to 20 degrees vertically; the left eye was moving in the range of -5 to five degrees laterally and -5 to 20 degrees vertically. The images of both eyes overlapped, and the patient no longer complained of diplopia (Figure 4c). The right eye of the control moved within the ranges of 0 degrees in the left-right direction and -5 to five degrees in the vertical direction, whereas the left eye moved within the ranges of -5 to 0 degrees in the left-right direction and -5 to five degrees in the vertical direction (Figure 4e).

Changes in motor function over time are presented in Table 3. Improvement was observed in all parameters.

**Table 3:** Changes in motor function over time SARA: Scale for the Assessment and Rating of Ataxia; FBS: Functional Balance Scale; TUG: Timed Up and Go Test; SWMT: Semmes-Weinstein Monofilament Test

Evaluation item	Before the start of training	Two weeks later	10 weeks later
SARA	14.5 points	－	11.5 points
Knee extensor strength (Right)	0.50 kgf/kg	－	0.64 kgf/kg
Knee extensor strength (Left)	0.61 kgf/kg	－	0.83 kgf/kg
FBS	14 points	21 points	33 points
TUG	Not testable	76 seconds	38 seconds
10 m walking time	Not testable	61 seconds	40 seconds
Hallux motion sense (Right)	1/5 trials	－	2/5 trials
Hallux motion sense (Left)	5/5 trials	－	5/5 trials
SWMT (Right)	4.56	－	4.56
SWMT (Left)	4.31	－	4.31

## Discussion

The study patient had strabismus and diplopia due to palsy of the right oculomotor nerve, right pulmonic nerve, and left abducens nerve. Patients with diplopia due to oculomotor nerve palsy, glenoid nerve palsy, and abducens nerve palsy after stroke often report gait disturbance as the most common problem in their daily living [16]. Thus, in this case, gait disturbance was predicted to be related to eye movement disorder.

Eye movement training to improve visual function has been developed for post-stroke patients, and its effectiveness has been demonstrated [17-19].

It has been reported that oculomotor rehabilitation can improve contraction and weakness of the extraocular muscles, increase the oculomotor range of motion [15], and prevent secondary changes such as shortening and atrophy of the extraocular muscles [20].

The right and left eyes rotate in the same direction by the same amount [21,22]. Therefore, the left and right eyes could look in the same direction and perceive the images as one. We speculate that eye movement training in the present patient expanded the range of motion of the eyes and enabled both eyes to follow each other jointly. One reason for this is that the correlation coefficient between the left and right eye movement angles of both eyes was close to one after eye movement training was completed. The correlation coefficients for the range of motion of both eyes in healthy individuals range from 0.97 to 0.99 or higher [21], and the correlation coefficient in our case was within this range. However, the correlation coefficient between the vertical eye movement angles of both eyes was high before the start of eye movement training but the slope of the regression line was 2.71. The left and right eyes moved in the same direction but in different amounts (the right eye moved one degree, whereas the left eye moved approximately three degrees). Additionally, the figure on the wall followed by both eyes was misaligned. After eye movement training, the slope of the regression line was close to one, and the overlapped figures on the wall were confirmed.

The left and right eye movements during walking were similar to those of healthy individuals; the left and right gazes overlapped, both eyes were able to see the center of the forward visual field, and the patient no longer complained of diplopia. These results suggest that eye movement training improved strabismus and diplopia, enabling the patient to understand the surrounding situation, which led to an improvement in his walking ability. However, it cannot be denied that all motor functions improved compared to those before the training and that this had an impact on the improvement in walking ability.

Limitations of this report and future issues

In this case, symptoms such as ataxia and sensory disturbance were observed in addition to eye movement disorder, and it is possible that the improvement in walking ability was due to factors other than eye movement improvement. Therefore, one must be cautious when concluding that improvements in walking ability are caused by eye movement training. It has also been reported that eye movement training can prevent shortening and atrophy of the external ocular muscles due to disuse [15] and cause plastic changes in the brain [23-25]; however, the lack of data to support this is a limitation and remains a challenge for the future.

## Conclusions

This study revealed that eye movement training is effective for visual function in stroke patients with visual impairment. Our results suggest that in addition to conventional motor training, eye movement training may be a way to improve walking ability in patients with stroke to obtain a stable gait.
